# Comparison of the Recycling Behavior of a Polypropylene Sample Aged in Air and in Marine Water

**DOI:** 10.3390/polym15092173

**Published:** 2023-05-03

**Authors:** Francesco Paolo La Mantia, Roberto Scaffaro, Marilena Baiamonte, Manuela Ceraulo, Maria Chiara Mistretta

**Affiliations:** 1 Dipartimento di Ingegneria, Università di Palermo, Viale delle Scienze, 90128 Palermo, Italy; francescopaolo.lamantia@unipa.it (F.P.L.M.);; 2 INSTM, Consorzio Interuniversitario Nazionale di Scienza e Tecnologia dei Materiali, Via Giusti, 9, 50121 Firenze, Italy

**Keywords:** recycling, degradation, marine water

## Abstract

During the processing and during their lifetime, polymers are subjected to several environmental stresses—thermomechanical, photo-oxidative, etc.—that can strongly modify their chemical and molecular structure and, consequently, their morphology. Reduction of the molecular weight and formation of double bonds and oxygenated groups are the main changes observed as a consequence of the degradation. As a result of these changes, the macroscopic properties are dramatically modified. These changes can have a relevant effect if the post-consumer plastic manufacts are recycled. In this work, a sample of polypropylene subjected to two different degradation histories—photo-oxidation in air and in marine water—is reprocessed two times in a mini twin-screw extruder in the same processing conditions. The effect of the thermomechanical degradation during the reprocessing is different. Indeed, the less severe degraded sample shows a higher degradation level during reprocessing because the shear stress is larger. This means that the thermomechanical degradation kinetics is larger in the less degraded samples. Nevertheless, the final properties of the recycled polymers are different because the properties of the photo-oxidized samples before reprocessing were very different.

## 1. Introduction

The recycling of post-consumer polymers depends mainly on their chemical and molecular structure [1,2,3,4,5] and on their morphology and composition in case of blends or composite [6,7,8,9]. The post-consumer polymers have been subjected to thermomechanical stress during the processing in the melt state and to different environmental stresses (temperature, ultraviolet rays, etc.) during their lifetime. On the other hand, the degradation undergone during the first processing can give rise to a more rapid degradation if the polymer is subjected to external stress such as the photo-oxidation or different processing conditions [10,11,12]. In this case, indeed, the presence of oxygenated groups or double bonds accelerates the degradation kinetics. The degradation gives rise to the reduction of molecular weight, the formation of oxygenated groups and double bonds and other structural and molecular changes. Moreover, changes of the morphology in semicrystalline polymers can also occur [6,7]. As the degradation depends on the initial chemical and molecular structure, due to these changes, the answer to the reprocessing processes in molten state should be different with respect to that of the same undegraded or a differently degraded polymer. As the degradation gives rise to polymers with lower molecular weight and with oxygenated groups, a study about the effect of the degradation on the recycling should investigate the influence of these two parameters on the processability and on the final properties of the reprocessed polymers. If temperature and shear stress are the driving force of the thermomechanical degradation during processing, the recycling in the same processing conditions of the same polymer but with different molecular weight should give different degradation kinetics as the viscosity of the different samples gives rise to different shear stress acting on the melt and then to a different driving force able to break the macromolecules. Moreover, the presence of oxygenated groups or vinyl bonds changing the energy necessary to break these bonds with respect to the –C–C– and –C–H– bonds present in the virgin polyolefins, such as polyethylene of polypropylene, can, in its turn, modify the degradation kinetics.

Papers investigating the effect of the molecular weight on the thermal degradation [13,14] and on the ultrasonic degradation [15,16] indicate a moderate effect of the molecular weight and, in particular, the cleavage of the macromolecules increases with increasing the molecular weight. In the papers [17,18,19,20,21], it was demonstrated that the polymer degraded more rapidly than for samples subjected only to processing or aging separately. Moreover, it was reported that reprocessing after aging caused a degradation greater than for those samples that were processed and then aged. An interesting way to evaluate the effect of the previous “life”, and then of the molecular structure on the recycling of post-consumer plastics, is the recycling of ocean-bound plastics. Indeed, the degradation of the polymers is certainly different than that undergone in air [22,23,24]. In particular, at the same degradation time, the level of degradation is lower than that observed in the same conditions, but in air; however, the previous papers discuss only the effect of the degradation in the sea water, without any comparison with the recycling of the same polymers degraded in air.

To our best knowledge, the effect of the degradation undergone during the lifetime in different environments but in the same conditions of external driving forces (temperature, UV irradiation, etc.) on the recycling of polymers, and then the effect of the molecular weight and of the presence of oxygenated groups on the thermomechanical degradation, has not been investigated.

In this work, the recycling behavior of a sample of polypropylene that degraded, at the same fixed time in two different conditions, has been investigated. The degradation has been carried out by photo-oxidizing the polypropylene in two different environments—air and marine water—as reported in our previous paper [25]. As reported in this last work, the photo-oxidation in marine water is less severe than in air under the same irradiation conditions because of the less availability of oxygen in marine water. The two degraded samples show different molecular weights and the presence of different number of oxygenated groups and this different chemical and molecular structure gives rise to different behaviors when they are reprocessed. In particular, the degradation during processing is more severe for the less degraded sample. However, the two-degradation kinetics are not so different, although the molecular weight and then the viscosities are very different. This behavior has been interpreted considering that the less degraded sample shows higher viscosity and then a higher value of the shear stress acting on the melt; however, on the other side, the presence of more labile bonds in the more severe degraded sample can compensate, at least in part, for the lower shear stress acting on this last sample.

This investigation can give many useful pieces of information when the recycling is carried out on post-consumer plastic manufacts with different level of degradation, including, for example, bottles and films degraded in different environments such as in air or in the sea.

## 2. Materials and Methods

### 2.1. Material, Degradation and Reprocessing

**Figure 1:**
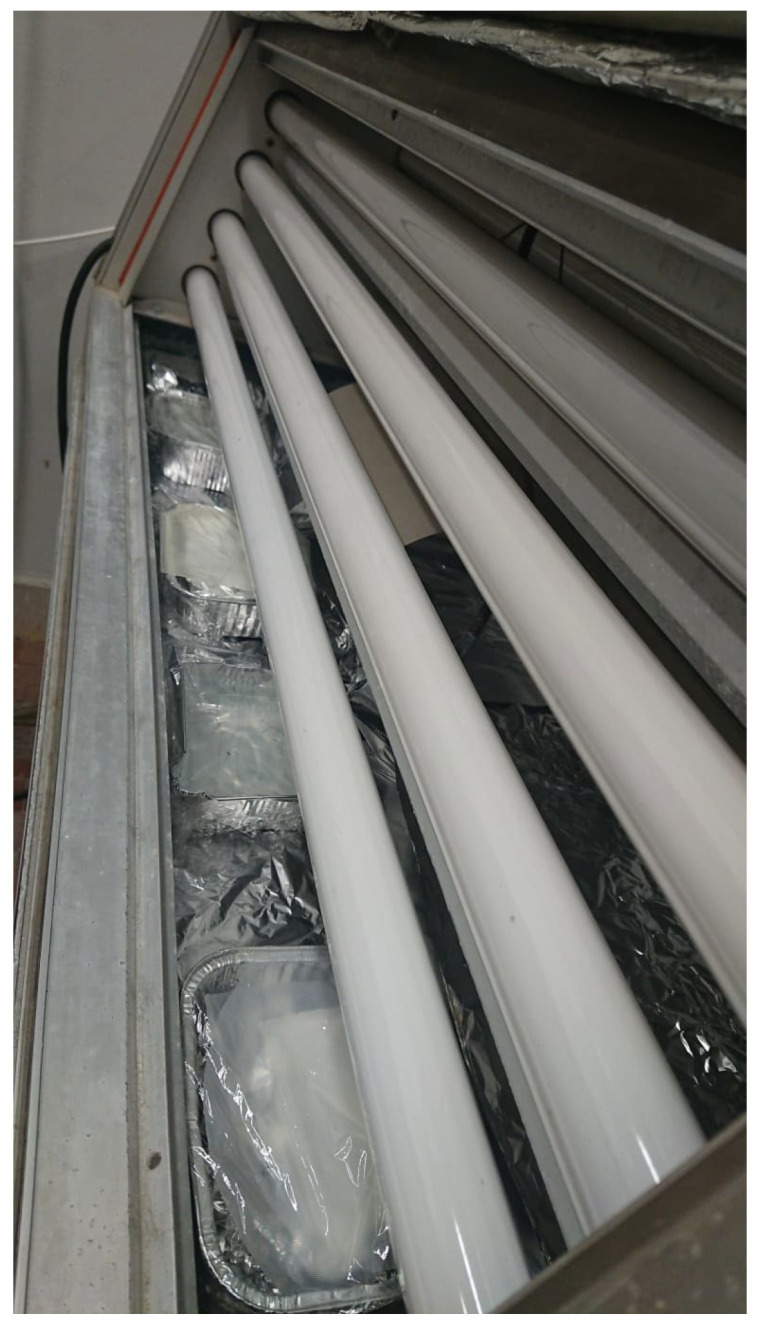
Picture of experimental setup.

The polypropylene (PP) used in this work is a random polypropylene copolymer Moplen RP34OH manufactured by LyondellBasell (LyondellBasell, Ferrara, Italy), having a melt flow index (MFI) of 1.8 g/10 min (230 °C/2.16 kg) and a density of 0.90 g/cm^3^. The photo-oxidation was carried out in a QUV at 40 °C for 192 h in air and in marine water, following the same procedure reported in [25]. The lamps were UVB 313 nm with a UV irradiation peak at 313 nm. As for the samples irradiated in marine water, the sheets were kept in aluminum trays immersed in water and covered by a film of PVC. The same arrangement was used for the samples irradiated in air. The trays were kept on the bottom of the QUV under the lamps. The distance of the samples from the lams was about 5 cm from the bottom lamp to about 22 cm from the top lamp. A picture of the experimental setup is reported in Figure 1.

The reprocessing of the samples photo-oxidized both in air and in marine water was performed in a laboratory conical mini-twin screw extruder (Minilab, Thermo Haake, Karlsruhe, Germany) at the temperature of 240 °C and at a rotational speed of 60 rpm. The degraded samples were extruded up to two times in the same conditions.

### 2.2. Characterizations

The FTIR-ATR spectra were recorded by using a Spectrum One spectrometer (Perkin-Elmer, Norwalk, CT, USA), equipped with integrated Spectrum One software. The spectra were obtained through 8 scans in the range 500–4000 cm^−1^. The spectra resolution was 4 cm^−1^. The specimens for the FTIR-ATR spectra were carefully wiped and dried before the measurement.

The rheological characterization was performed by using a rotational rheometer ARES G2 (TA Instruments, New Castle, DE, USA) with parallel plate, at the temperature of 190 °C in the frequency range of 0.1–100 rad/s. The strain was 5% for all the tests. The diameter of the specimens was 25 mm.

The mechanical characterization was carried out in tensile mode using an Instron (Instron, High Wycombe, PA, USA) mod. 3365 universal machine at a crosshead speed of 1 mm/min until a deformation of 3%, and then at a crosshead speed of to 100 mm/min until final rupture. The dimensions of the specimens for the tensile tests were 90 × 10 mm. Seven replicates for each measurement were performed, in order to obtain statistically relevant results. The reproducibility of the results was good (max ± 8%).

The samples used for all the tests, about 0.7 mm thick, were obtained by compression molding in a laboratory Carver press (Carver, Wabash, IN, USA) at 190 °C and at a mold pressure of 300 psi for about 2 min.

## 3. Results

### 3.1. Characterization of the Degraded Samples

**Figure 2:**
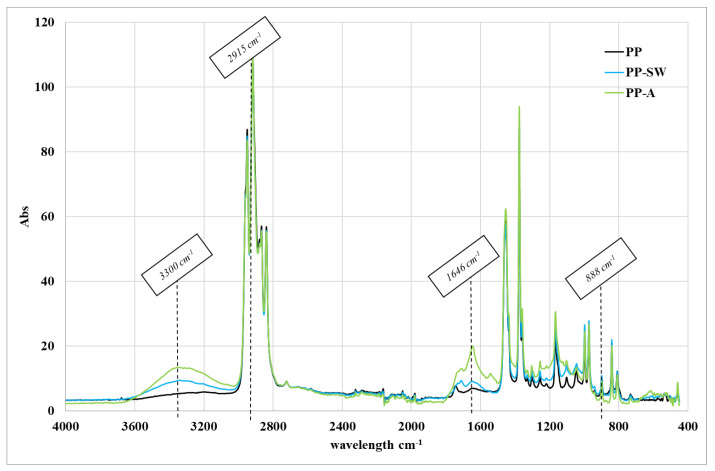
ATR spectra of the virgin PP, PP degraded 192 h in air, and PP degraded 192 h in marine water (PP-SW).

Figure 2 reports the ATR spectra of the samples investigated in this work, PP degraded 192 h in air (PP-A) and PP degraded 192 h in marine water (PP-SW) compared with the spectra of the virgin polymer. The aged polymers show the formation of oxygenated groups mainly in two different regions centered at about 1720 and 3340 cm^−1^. The first band is attributed to the formation of ketone groups, while the second band is attributed to the formation of hydroxyl groups. Moreover, a slight rise of the spectra in the range of 1600–1700 cm^−1^, attributable to the formation of vinyl bonds and carboxyl groups [26], was also observed. The more oxygenated sample was the sample photo-oxidized in air. This behavior was interpreted [25] as a result of the lower content of oxygen available in the marine water.

**Figure 3:**
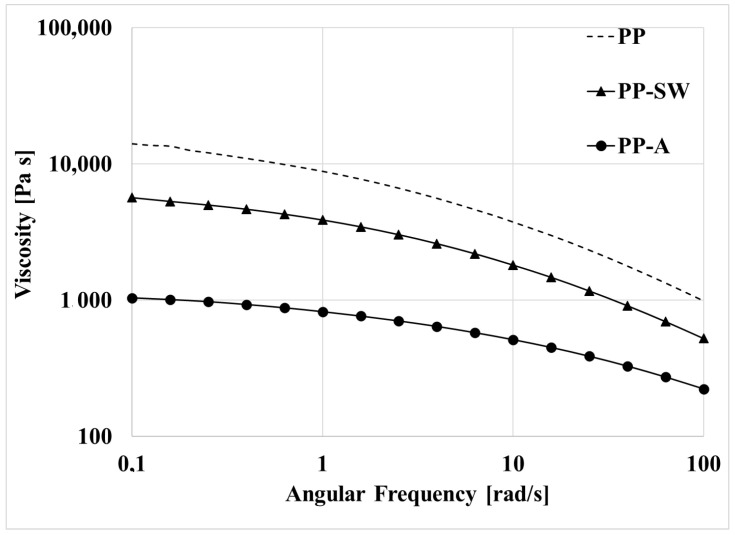
Flow curves of the virgin PP and of the photo-oxidized PP-A and PP-SW samples.

The flow curves of the same three samples are reported in Figure 3. The flow curves of the two degraded samples and of the virgin sample are, of course, below that of the virgin polymer and the sample photo-oxidized in air shows the lower values of viscosity. The sample photo-oxidized in air shows a reduction of the Newtonian viscosity of more than one decade. Moreover, the flow curves of the photo-oxidized samples show a less pronounced non-Newtonian behavior. The lowest viscosity of the PP-A sample means that this sample presents the lowest molecular weight.

The reduction of the molecular weight can be evaluated by considering that the Newtonian viscosity is [27]:

η_0_ = kMw^3.4^ (1)

where η_0_ is the Newtonian viscosity, K a constant and Mw the weight average molecular weight. The dimensionless molecular weight, $\bar{Mw}$, of the sample at a given irradiation time is:

(2) $${\bar{Mw}(t)=(\eta_{0}(t)/\eta_{0}(0))}^{1/3.4}$$

where η_0_(t) is the Newtonian viscosity at a given irradiation time, t, and η_0_(0) is the Newtonian viscosity of the virgin sample. The molecular weight is reduced only to about 34% of the initial value for the sample irradiated in air and to about 76% of the initial value for the sample irradiated in marine water. The lower degradation kinetic for the sample irradiated in marine water is due, as already reported [24], to the lower content of oxygen available in marine water.

In Table 1 the ultimate mechanical (tensile) properties, tensile strength (TS) and elongation at break (EB) of the same investigated samples are reported.

As expected, the decrease in both tensile strength and elongation at break is dramatic only for the more degraded sample and quite modest for the less degraded sample. In particular, the elongation at break that is very sensible to the changes of the molecular structure and morphology is strongly reduced only in the sample photo-oxidized in air.

### 3.2. Characterization of the Reprocessed Samples

**Figure 4:**
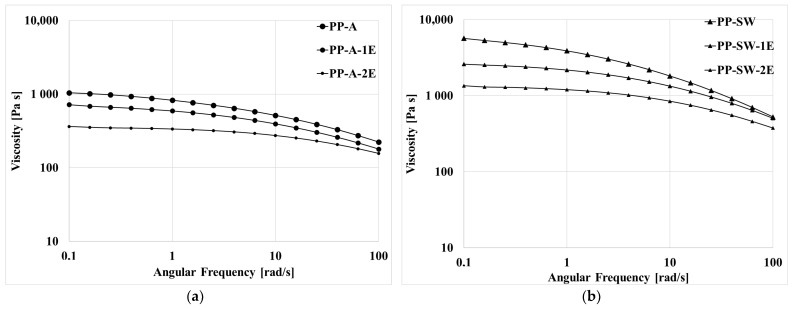
Flow curves of the recycled samples: (**a**) PP-A, (**b**) PP-SW.

In Figure 4a,b the flow curves of the two recycled samples are reported. Of course, the viscosity decreases with the number of extrusions for both samples, showing the action of the thermomechanical stress that is able to break the macromolecules during both the two passages in the mini twin-screw extruder. The decrease in the Newtonian viscosity is larger for the PP-SW sample, which is clear evidence of a more severe thermomechanical degradation for this last sample. Of course, this is due to the higher viscosity of this sample that generates larger shear stress acting on the melt for this last sample with respect to the PP-A. Moreover, the non-Newtonian effect becomes less pronounced with increasing the reprocessing steps. The decrease in the viscosity is certainly relevant due to the high values of the shear stress in the twin-screw extruder.

Although these high shear stress values are not typical of the stress encountered in single-screw extruders, these processing conditions are useful to magnify the thermomechanical degradation and the influence of the different molecular and chemical structure on the recycling.

The values of the tensile strength and elongation at break of the sample reprocessed one and two times in the twin-screw extruder are reported in Table 2.

**Figure 5:**
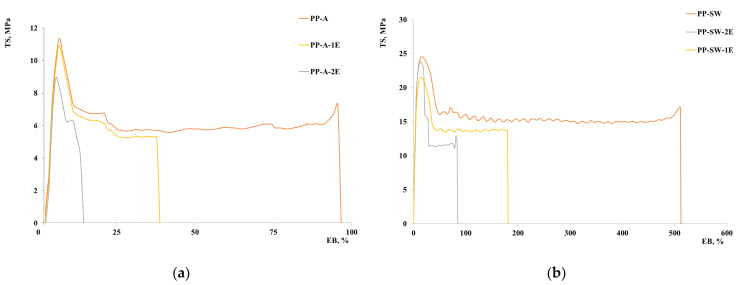
Stress–strain curves: (**a**) PP-A, (**b**) PP-SW.

Additionally, for tensile strength and elongation at break a decrease is observed with increasing the number of extrusions. A remarkable reduction of elongation at break and tensile strength is mainly observed for the sample PP-SW. However, the decrease in the tensile strength is about 43% after two extrusions for the PP-A and about 47% for PP-SW, and the decrease in the elongation at break for PP-A is about 85% and about 88% for the sample PP-SW. It is worth mentioning, then, that the kinetic of degradation seems similar for both samples, as it will be discussed in the following, but the absolute values of the mechanical properties are very different because the properties of the aged samples are very different. Figure 5 shows the stress–strain curves.

**Figure 6:**
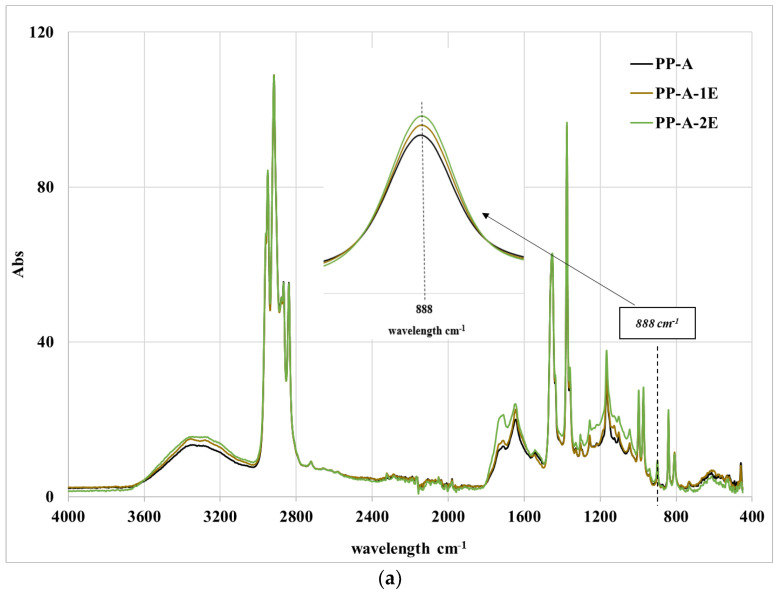

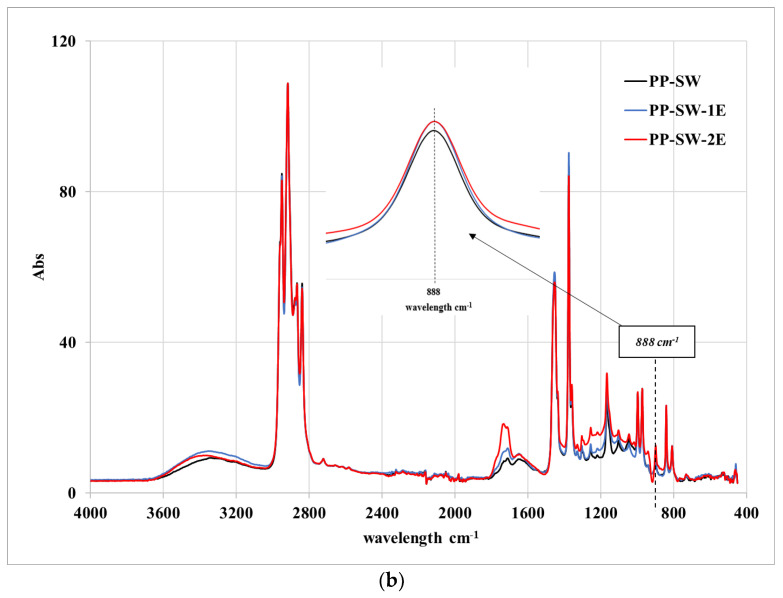
ATR spectra of all the reprocessed samples: (**a**) PP-A; **(b**) PP-SW.

The ATR spectra of all samples are reported in Figure 6a,b. The spectral bands at 3300–3400 and 1600–1700 cm^−1^ increase with the number of extrusions. The spectral band is centered at about 1720 cm^−1^, but a significant increase is also observed in the spectral range of 1600 and 1700 cm^−1^ relative to the formation of vinyl and carboxylic acid. However, the formation of new oxygenated groups is quite low for both bands due, presumably, to the low presence of oxygen in the mini-extruder and the low residence times. A small increase of the spectra at about 888 cm^−1^ is also observed, as represented in Figure 6a,b, due to the formation of vinylidene compounds.

## 4. Discussion

**Figure 7:**
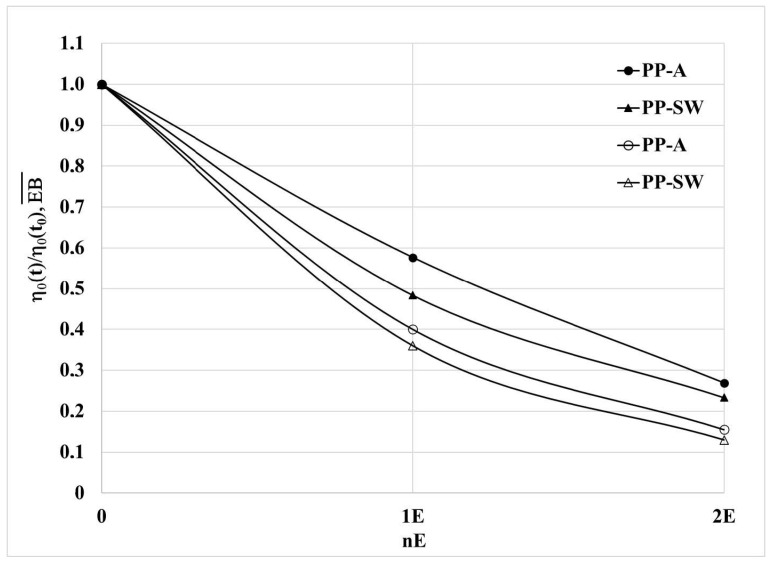
Dimensionless values of the Newtonian viscosity and of the elongation at break as a function of the number of extrusions. Open symbols refer to elongation at break; closed symbols refer to viscosity.

In order to investigate the effect of the different molecular structure on the degradation kinetic, the rheological and mechanical results have been normalized to put in evidence the kinetic of degradation. In Figure 7, the dimensionless values of the Newtonian viscosity of the reprocessed samples are reported as a function of the number of extrusions. The dimensionless values are calculated as the ratio between the value of the Newtonian viscosity after one and two extrusions by the value of the photo-oxidized, unprocessed sample. In Figure 7, 0 means the unprocessed samples, while 1 and 2 refer to one and two reprocessing steps.

It is evident that the decrease in the viscosity, and then in the molecular weight, is faster for the sample degraded in marine water. It is, however, worth mentioning that the velocity of the decay of the viscosity is high in the first extrusion, and then the slope of the curve decreases for both samples and the slopes of the curves of the two samples become similar. This behavior can be interpreted considering that the driving force of the thermomechanical degradation is the shear stress proportional to the viscosity that decreases, and the viscosity of the PP-SW sample approaches that of the sample degraded in air. The same comments can be made for the dimensionless values of the elongation at break reported in the same figure. The elongation at break is the mechanical property more dependent on the variations of molecular structure of the polymer.

The mechanical stress acting on the melt is the shear stress in the processing conditions, i.e., the shear rate, γ, by the viscosity in the processing conditions, η,

Τ = η ∗ γ.(3)

**Figure 8:**
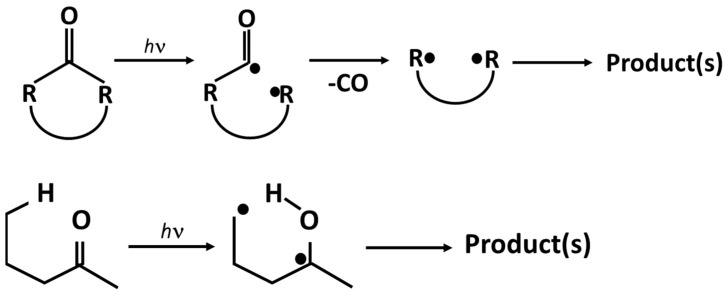
NORRISH REACTIONS.

The shear rate is, of course, the same in all the tests because the screw speed is the same, while the viscosity is different for the two samples (see Figure 3): higher for the sample PP-SW and lower for the sample degraded in air. This means that the thermomechanical stress is low for PP-A and higher for PP-SW and the consequent degradation is higher for PP-SW and low for PP-A. However, the difference in the degradation kinetic is not dramatically different and seems lower than that expected on the basis of the very different viscosities of the two unprocessed samples and then on the basis of the different shear stress at which the polymers are subjected. A possible interpretation of this behavior can be correlated with the different chemical structure of PP-A and PP-SW. Indeed, the first sample presents an initial number of oxygenated groups certainly higher than that observed in the PP-SW sample. The energy bonds of the generic C–C=O bonds are lower than that of the other C–C bonds and, in analogy with the photo-oxidation, Norrish reactions can be invoked to interpret this phenomenon. The degradation in the PP-SW sample is mainly due to the high thermomechanical stress. However, in the PP-A sample the lower shear stress is efficient because of the Norrish reactions, Figure 8, due to the highest presence of oxygenated groups. The two samples show similar degradation kinetics, although they are subjected to different driving thermomechanical forces because of the different energy necessary for the cleavage of the different carbon–carbon links.

## 5. Conclusions

The properties of recycled polymers depend mainly on the molecular and chemical structure of the reclaimed post-consumer plastic manufacts. To our best knowledge, no specific papers have dealt with the effect of the chemical and molecular structure of the post-consumer plastics on the recycling operations and on the final properties of these polymers. In this work a polypropylene sample photo-oxidized in two different environments, air and marine water, have been reprocessed in the same processing conditions of temperature and UV irradiation in order to evaluate the effect of the level of the initial degradation, and then of the previous life, on the thermomechanical degradation during reprocessing and on the final properties of the recycled material. The less degraded sample, PP-SW, having larger molecular weight and viscosity, shows a higher degradation kinetic and a larger level of thermomechanical degradation because of the higher viscosity of this sample that gives rise to higher stress on the melt that is able to break the macromolecules. However, the least amount of energy needed to break lower energy bonds of the carbon bonds with oxygenated carbon groups in the more degraded sample implies an increase of the degradation kinetics of this last PP-SW sample. In short, the lower molecular weight decreases the thermomechanical degradation but, on the contrary, the presence of oxygenated groups increases, through the Norrish reactions, the thermomechanical degradation. Finally, while the degradation kinetic during processing seems similar for both samples, the absolute values of the viscosity and of the ultimate mechanical properties are, instead, very different because the same properties were very different in the aged samples before processing. In this investigated case, the polymers aged in marine water shows better properties after the recycling processing than those shown by the polymers aged in air. This behavior allows us to consider, for example, that ocean-bound post-consumer plastics can be mechanically recycled in apparatuses and in reprocessing conditions similar to those used for the mechanical recycling of all the other post-consumer plastic manufacts.

## Figures and Tables

**Table 1:** Tensile strength and elongation at break.

Sample	TS [MPa]	Dev. St.	EB [%]	Dev. St.
PP	22.2	0.7	626	41.8
PP-A	7.4	1.1	93	6.6
PP-SW	20.9	0.7	540	63.4

**Table 2:** Tensile strength and elongation at break of the degraded samples (0) and of the samples reprocessed one and two times (nE).

Sample	nE	TS [MPa]	Dev. St.	EB [%]	Dev. St.
	0	7.4	1.1	93	6.6
PP-A	1	5.2	0.5	38	6.5
	2	4.2	0.9	14	1.7
	0	20.8	0.7	540	63.4
PP-SW	1	14.1	1.1	196	29.9
	2	11.1	1.7	72	1.4
